# Modeling the relationship between depression in people with cancer and perceived stress, with the mediating role of eating problems, sexual satisfaction, emotion regulation and self-compassion

**DOI:** 10.3389/fpsyg.2024.1281347

**Published:** 2024-04-10

**Authors:** Reihaneh Moniri, Banafsheh Gharraee, Komeil Zahedi Tajrishi

**Affiliations:** Department of Clinical Psychology, School of Behavioral Sciences and Mental Health (Tehran Institute of Psychiatry), Iran University of Medical Sciences, Tehran, Iran

**Keywords:** depression, cancer, perceived stress, eating problems, sexual satisfaction, emotional regulation, self-compassion

## Abstract

**Aim:**

With the rising prevalence of cancer and the adverse physical and psychological experiences endured by affected individuals, this study aims to establish a model illustrating the relationship between depression in people with cancer and perceived stress. Additionally, it examines the mediating influence of eating problems, sexual satisfaction, emotional regulation, and self-compassion.

**Method:**

This study employs a descriptive-correlational research method, utilizing structural equation analysis (modeling) to explore the role of mediators. The research sample comprised 200 individuals diagnosed with cancer, selected based on predetermined inclusion and exclusion criteria. Participants completed Beck’s 13-item depression questionnaire, a 10-item perceived stress questionnaire, an 8-item appetite measurement questionnaire, a 25-item sexual satisfaction questionnaire, a 10-item emotion regulation questionnaire, and a 12-item compassion questionnaire. The data were subsequently analyzed using SPSS-24 and Lisrel 80/8 software.

**Findings:**

The research findings indicate a significant positive relationship between perceived stress and depression in people with cancer. Furthermore, eating problems exhibited a mediating role in the relationship between perceived stress and depression, with a direct effect coefficient of (*t* = 0.28, *ß* = 0.02). However, the path from perceived stress to depression, mediated by sexual satisfaction, was found to be statistically insignificant, with a standard coefficient of 0.01 at the *p* < 0.05 level. Emotion regulation demonstrated a direct effect coefficient of (*t* = −3.52, *ß* = –0.33) on depression. Likewise, self-compassion exhibited a direct effect coefficient of (*t* = −3.08, *ß* = –0.28) on depression, while the path from perceived stress to depression was mediated by self-compassion, with a standard coefficient of 0.12 at the *p* < 0.05 level.

**Conclusion:**

In conclusion, this study sheds light on the interplay between depression and perceived stress in individuals with cancer, revealing a significant positive association. Eating problems emerged as a mediating factor, directly influencing the manifestation of depressive symptoms. However, the mediation pathway through sexual satisfaction was found to be statistically insignificant. Emotion regulation and self-compassion were identified as influential factors, with direct effects on depression and self-compassion also serving as a mediator in the relationship between perceived stress and depression. The findings emphasize the importance of targeted interventions addressing eating problems, enhancing emotion regulation, and fostering self-compassion to alleviate the impact of depression and perceived stress in individuals facing cancer-related challenges. Further research is encouraged to refine and expand upon these insights, advancing holistic care for this population.

## Introduction

Cancer represents a leading cause of mortality worldwide. According to the World Health Organization, the number of new cancer cases in 2020 reached 19 million, with over 10 million resulting in death. In essence, one out of every six deaths is attributable to cancer. The most prevalent types of cancer include breast cancer in women, as well as lung, colon, prostate, skin, and stomach cancer (Bray et al., 2018). These statistics are equally alarming within our country, with current registered cases numbering 112,000, projected to increase by 43% to 160,000 cases by the year 2026, according to Iranian researchers. Stomach cancer and breast cancer among men and women, respectively, exhibit the highest prevalence, a trend expected to persist until 2026 (Roshandel et al., 2021). The impact of cancer manifests in various physical and psychological aspects, including depression, anxiety, quality of life, sexual function, fatigue, coping and adaptation skills, and body image (Saeedi et al., 2019). Psychological distress and psychiatric disorders commonly afflict cancer patients, severely compromising their quality of life. Moreover, psychological disorders can engender behaviors that heighten the risk of cancer (Miovic and Block, 2007; Sartorius et al., 2014).

Among these psychological issues, mood disorders, particularly depression, prevail as the most common conditions associated with cancer (Kissane et al., 2004). Recent studies have underscored the augmented risk of depression in individuals experiencing heightened perceived stress (Alagizy et al., 2020).

Notably, cancer patients, exhibit perceived stress alongside depression and anxiety (Cristóbal-Narváez et al., 2022). Prior investigations have examined the relationship between depression in cancer patients and perceived stress, employing mediators such as self-esteem (Lee et al., 2013) and mental adjustment (Li et al., 2015). Consequently, interventions aimed at addressing perceived stress can effectively ameliorate depression in people with cancer, thus improving their overall well-being (Hur and Song, 2008). Perceived stress levels in cancer patients also significantly correlate with their self-care behaviors, including crucial aspects such as healthy eating habits (Abdollahi et al., 2020).

Healthy eating habits, encompassing the regulation of appetite and the resolution of eating problems, constitute a pivotal component of self-care behaviors in individuals grappling with depression (Mischoulon et al., 2011; Lee et al., 2013). Multiple studies have demonstrated a substantial association between perceived stress and eating disorders (Asghari et al., 2017), as well as eating behaviors (Asberg and Wagaman, 2010; Roy et al., 2021).

The impact of stress on sexual components has been explored, elucidating the connection between stress and sexual performance (Grønli et al., 2005), behaviors (Tavares et al., 2019), and well-being (Lin et al., 2022). Women with cancer, especially those affected in their sexual organs, may encounter various sexual problems that can persist even a year after recovery (Hamilton and Meston, 2013). Depression also exhibits a pronounced association with sexual problems, including sexual dissatisfaction resulting from fatigue and diminished pleasure (Peleg-Sagy and Shahar, 2013; Din et al., 2019). Consequently, this aspect warrants further investigation.

Emotion regulation emerges as a significant variable, correlating notably with perceived stress and depression in cancer patients (Boyle et al., 2017). Given the significant relationship between perceived stress levels and the employment of emotion regulation strategies (Zahniser and Conley, 2018), as well as the connection between emotion regulation and depression, this component holds promise as a mediator between stress perception and depression in cancer patients (Joormann and Stanton, 2016).

Self-compassion, encompassing the three subscales of self-compassion versus self-blame, human commonality versus isolation, and mindfulness versus identification, functions as a protective factor against depression in individuals diagnosed with cancer (Zhu et al., 2019). Notably, this component exhibits a significant relationship with perceived stress (Homan and Sirois, 2017), qualifying it for examination as a mediator between perceived stress and depression in cancer patients.

**Figure 1:**
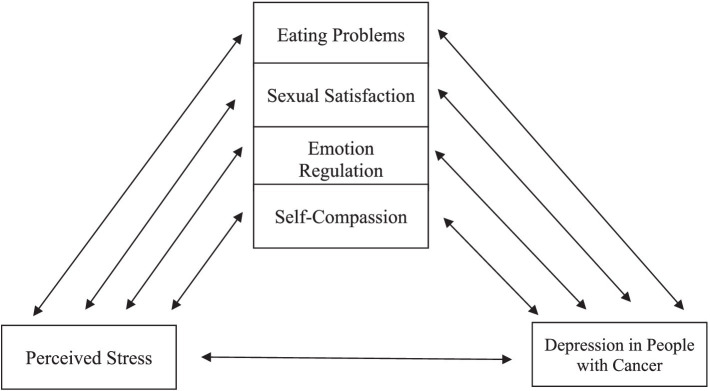
Assumed model.

Despite numerous studies exploring various factors contributing to depression in cancer patients, a comprehensive model has yet to be presented. This research seeks to establish a model that systematically examines the relationships between depression in people with cancer and perceived stress. To achieve this, we will consider the mediating effects of eating problems, sexual satisfaction, emotional regulation, and self-compassion. The proposed model is depicted in Figure 1.

Research questions include:

1. Can eating problems play a mediating role in the relationship between depression in people with cancer and perceived stress?
2. Can sexual satisfaction play a mediating role in the relationship between depression in people with cancer and perceived stress?
3. Can emotion regulation play a mediating role in the relationship between depression in people with cancer and perceived stress?
4. Can self-compassion play a mediating role in the relationship between depression in people with cancer and perceived stress?
5. Does the assumed model have a good fit?

## Method

The current research utilized a descriptive-correlational design to examine the mediating role of eating problems, sexual satisfaction, emotion regulation, and self-compassion in the relationship between depression and perceived stress in cancer patients. Structural equation analysis (modeling) was employed for data analysis.

To ensure the inclusion of relevant variables, the study focused on cancer patients with blood, bone, liver, lung, breast, mouth, Lymphoma, skin, and thyroid cancer. This targeted sampling approach was chosen due to the significant impact of certain cancer types, such as stomach and intestine cancer, on variables like sexual satisfaction and eating problems (Kline, 2023). According to Klein’s studies, the required sample size was 200 people for the significance of the studies using the modeling method. The population of this research included people with cancer undergoing treatment in hospitals and clinics in Tehran in 2023.

The study utilizes various instruments, namely the Beck Depression Questionnaire, Perceived Stress Scale (PSS), the Council on Nutrition Appetite Questionnaire (CNAQ), Larson’s Sexual Satisfaction Questionnaire, ERQ Questionnaire, and the Short Form of the Self-Compassion Questionnaire. Subsequent paragraphs will provide detailed discussions on each of these instruments.

After obtaining the necessary permits from the Iran University of Medical Sciences and Health Services and receiving the code of ethics, the oncology department of Hazrat Rasool Akram Hospital and Shahid Hasheminejad Kidney and Urinary Tract Hospital in Tehran was referred to collect data. At this stage, the paper-pencil version of the questionnaires was used to collect data, and by visiting the relevant hospitals and explaining the research objectives and making the necessary arrangements, cancer patients, both inpatients and outpatients to receive chemotherapy drugs (to injection) were invited to participate in the study. The objectives of the research, the condition of informed consent and the possibility of withdrawing from the study were explained to them, and in case of written consent, the questions were provided to them. At first, explanations about the questionnaire questions and how to complete them were provided to the patients, they were assured that the names will not be written on the questionnaire and only the researcher will have access to the data from the questionnaire. At the end, the patients were requested to complete the questions carefully and not to leave any question unanswered. Eleven people withdrew from the research due to personal reasons and 12 questionnaires were incompletely completed, which were excluded from the analysis, and the final sample consisted of 200 subjects.

Statistical data analysis involved the use of both descriptive and inferential analysis methods. SPSS-24 software was utilized to analyze mean, standard deviation, frequency percentage, and correlation. Additionally, Lisrel 80.8 software was employed for structural equation modeling.

### Instruments

#### Depression questionnaire

The Beck depression questionnaire, initially developed by Beck and colleagues in 1961, was utilized. The short version of this questionnaire consists of 13 questions graded on a four-point Likert scale ranging from 0 to 3. The total score ranges from 0 to 39, with negative affect toward oneself measured by questions 1, 2, 3, 4, 5, 6, 7, and 10, and unpleasantness assessed through questions 7, 8, 9, 11, 12, and 13. This questionnaire demonstrates acceptable internal reliability, with Cronbach’s alpha coefficients of 0.86 among psychiatric patients and 0.81 for non-psychiatric patients. Furthermore, it exhibits a satisfactory correlation with the Hamilton Depression Rating Scale (HRSD) (Beck et al., 1988). The Persian version of the questionnaire, standardized by Ghasemzadeh et al., showcases a high internal consistency score of Cronbach’s alpha 0.87 and a notable pre-test and post-test validity with *r* = 0.74 (Ghassemzadeh et al., 2005).

#### Perceived stress questionnaire

The Perceived Stress Scale (PSS), introduced by Cohen et al. in 1983, was employed. This questionnaire comprises 16 items graded on a five-point Likert scale ranging from 0 to 4. It measures two main subscales: perceived self-efficacy and perceived helplessness. The internal consistency coefficient reported in Cohen’s study for each subscale and the overall score ranged from 0.84 to 0.86 (Cohen et al., 1983). In the Persian version, standardized by Safaei and Shokri, the internal consistency coefficients for perceived self-efficacy, perceived helplessness, and the overall score of perceived stress were Cronbach’s alpha 0.80, 0.60, and 0.76, respectively (Safaei and Shokri, 2014).

#### Eating problems questionnaire

The Council on Nutrition Appetite Questionnaire (CNAQ) was utilized, developed by Wilson et al. in 2005. This measurement scale includes eight questions with five Likert options. Scores range from 8 (worst) to 40 (best), with higher scores indicating better nutritional status. The questionnaire demonstrates high similarity with a Cronbach’s alpha coefficient of 0.72 (Wilson et al., 2005). In the standardized Persian version by Sajadi et al., the questionnaire exhibits acceptable homogeneity with a Cronbach’s alpha coefficient of 0.5 (Sajadi Hezaveh et al., 2023).

#### Sexual satisfaction questionnaire

The sexual satisfaction questionnaire by Larson was utilized, consisting of 25 questions, with 13 negative and 12 positive items. Participants responded using a five-option Likert method. Scores ranged from 25 to 125, where scores below 50 indicated no sexual satisfaction, 51–75 indicated low satisfaction, 76–100 indicated moderate satisfaction, and scores above 100 indicated high satisfaction. The internal consistency was reported as alpha 0.91 (Hudson et al., 1981; Larson et al., 1998). In the Persian version standardized by Bahrami et al., the internal consistency was calculated using Cronbach’s alpha, yielding a value greater than 0.7 (Bahrami et al., 2016).

#### Emotion regulation questionnaire

The ERQ questionnaire was developed by Gross and John in 2003, consisting of 10 questions that measure two subscales: cognitive reappraisal (six items) and expressive suppression (four items). Participants responded on a seven-point Likert scale, with higher scores indicating better emotional regulation ability. The internal correlation for the cognitive reappraisal subscale was 0.79, while for expressive suppression, it was 0.73 (Gross and John, 2003). In the Persian sample of this questionnaire, Bigdeli et al. reported Cronbach’s alpha coefficients of 0.83 for cognitive reappraisal and 0.79 for expressive suppression (Bigdeli et al., 2013).

#### Self-compassion questionnaire

The self-compassion questionnaire (short form) consisted of 12 questions graded on a five-point Likert scale. It measured three subscales: self-compassion against self-blame, human commonality against isolation, and mindfulness against identification. The retest reliability for this scale was reported as 0.92 (Raes et al., 2011). The Persian version, standardized by Khanjani et al., reported alpha coefficients of 0.79 for the total scale and 0.68, 0.71, and 0.86 for the subscales of self-compassion against self-blame, human commonality against isolation, and mindfulness against extreme identification, respectively (Khanjani et al., 2016).

## Results

In this comprehensive study, a diverse group of 200 individuals spanning a wide range of ages, from 19 to 69 years, actively took part. The participants, characterized by an average age of 49.64 and a standard deviation of ±11.66, were carefully selected to ensure a representative sample. To provide further insights into the demographics, please refer to Table 1, which showcases the various characteristics of the research sample.

**Table 1:** Demographic characteristics.

		Frequency	Percentage
Gender	Female	122	61
	Male	78	39
Marital status	Single	20	10
	Married	158	79
	Others (divorced, divorced, widowed)	22	11
Degree of education	Undergraduate	95	47.5
	Diploma	73	36.5
	Bachelor	28	14
	Master	4	2
Job status	Employee	41	20.5
	Housewife	107	53.5
	Unemployed	23	11.5
	Student	2	1
	Retired	27	13.5
Inpatient status	Inpatient	98	49
	Outpatient	102	51
Family history of cancer	Yes	63	31.5
	No	137	68.5
Cancer type	Leukemia	16	8
	Breast	41	20.5
	Liver	41	20.5
	Bone	6	3
	Mouth	8	4
	Lung	5	2.5
	Skin	22	11
	Lymphoma	7	3.5
	Thyroid	54	27

### Descriptive results

Table 2 shows the minimum, maximum, mean and standard deviation of the scores of the research variables.

**Table 2:** Minimum, maximum, average and standard deviation.

Research variables	Sample size	Minimum	Maximum	Mean	Standard deviation
Depression	200	0	31	8.15	6.79
Perceive stress	200	12	32	19.83	4.22
Sexual satisfaction	200	46	121	81.99	15.83
Self-compassion	200	19	59	38.54	8.20
Eating problems	200	10	36	25.76	4.96
Emotion regulation	200	10	66	44.46	10.46

### Inferential results

In order to thoroughly validate the inferential findings, a comprehensive assessment of the path analysis defaults was conducted. Moving forward, the hypotheses and research questions were subjected to rigorous testing using both Pearson’s moment correlation coefficient tests and path analysis. To ensure the robustness of the analyses, various diagnostic tests were performed, including evaluations of univariate and multivariate normality assumptions, identification of potential multiple collinearity issues, examination of outliers, and scrutiny of missing data. Specifically, the assumption of univariate normality was assessed using the Smirnov Kolmograph test, and the results were meticulously analyzed. Table 3 presents the z statistic values obtained from this test, revealing that none of the calculated Smirnov Kolmograph test statistics reached significance (*p* > 0.05). This compelling outcome conclusively supports the notion that the variables’ scores follow a normal distribution, thereby affirming the appropriateness of employing parametric tests for subsequent analyses.

**Table 3:** The results of Smirnov Kolmograph test.

Variables	Kolmograph-Smirnov		
	Statistic	Df	Sig
Depression	0.073	200	0.052
Perceive stress	0.086	200	0.043
Sexual satisfaction	0.103	200	0.031
Self-compassion	0.067	200	0.053
Eating problems	0.072	200	0.052
Emotion regulation	0.099	200	0.039

In this research, the assumption of normality of several variables was thoroughly evaluated by calculating Merdia’s coefficient index. The resulting coefficient value was 2.362 for the hypothetical model. Taking into consideration that the critical value of the t for this coefficient is less than 2.58, it can be confidently concluded that the multivariate normality assumption holds true. This finding adds strength to the validity of the research.

Multiple collinearities, referring to the presence of high correlations between obvious variables, were meticulously examined. One of the widely accepted approaches to assess multiple collinearities is by scrutinizing the correlation matrix among these variables. A comprehensive analysis of the correlation matrix clearly indicated the absence of any significant multiple collinearities among the variables under investigation. The correlation coefficients for the hypothetical model ranged from 0.01 to 0.54. It is noteworthy that correlation coefficients exceeding 0.85 tend to hinder the accurate estimation of the model due to the problem of multiple collinearities. Thus, the assumption of the non-existence of multiple collinearities holds true, further ensuring the reliability of the study.

To identify univariate outlier data for the obvious variables, a frequency table and box plot were employed. Additionally, multivariate outlier data were identified by calculating Mahalanobis distances for each individual. Consequently, one participant was excluded from the analysis. Moreover, Table 4 displays the correlation matrix of the research variables, providing a comprehensive overview of the relationships among them.

**Table 4:** Correlation matrix.

**Variable**	**Depression**	**Perceived stress**	**Sexual satisfaction**	**Self-compassion**	**Eating problems**	**Emotion regulation**
Depression	1					
Perceive stress	0.18*	1				
Sexual satisfaction	−0.18*	−0.01	1			
Self-compassion	−0.54**	−0.23**	0.19**	1		
Eating problems	−0.30**	−0.30**	0.32**	0.30**	1	
Emotion regulation	−0.41**	−0.09	0.02	0.36**	0.13	1

**p < 0.01; *p < 0.05.

#### Question 1: Can eating problems play a mediating role in the relationship between depression in people with cancer and perceived stress?

**Figure 2:**
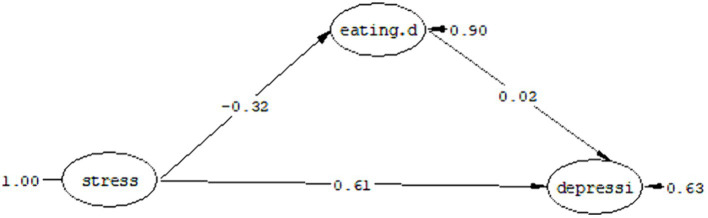
Mediation model of eating problems in the relationship between perceived stress and depression.

The fit indices as a result of the test of the proposed model are shown in Table 5, which shows that the proposed model has a good fit. As you can see in Figure 2, perceived stress has a direct effect (*t* = 0.32, *ß* = –3.79) on eating problems. The coefficient has a direct effect (*ß* = 0.61, *t* = 0.61) on depression. Also, eating problems have a direct effect on depression (*ß* = 0.28, *t* = 0.02). Considering that in the structural model, the significance of the path coefficient is determined using the value of t, if the value of t is more than 1.96, the relationship between the two structures is significant.

**Table 5:** Fit indices of the mediation model of eating problems in the relationship between perceived stress and depression.

**Fit indices**	**X** ^**2**^ **/df**	**CFI**	**IFI**	**NFI**	**NNFI**	**RFI**	**RMSEA**
	2.78	0.89	0/90	0/90	0.88	0.91	0.08

Table 6 shows the standardized coefficients, limits of the Bootstrap test and the estimation error of the indirect path (the mediation model of eating problems in the relationship between perceived stress and depression).

**Table 6:** Indirect path coefficients of the mediation model of eating problems in the relationship between perceived stress and depression.

**Indirect path**	**Indirect beta**	**Number of sample reproductions**	**Bootstrap value**		**Estimation error**
			**Highest**	**Lowest**	
Mediating role of eating problems in the relationship between perceived stress and depression	0.08	1,000	0.13	0.03	0.02

In the present study, the Bootstrap test was used to evaluate the mediation relationship. Bootstrap provides the most powerful and logical method to obtain indirect effects. As a result of this method, if the highest and lowest limits for the mediating path are of the same sign (both positive or both negative) or the zero value is not placed between these two limits, then the indirect causal path is significant. As you can see in Table 6, the highest and lowest limits of the Bootstrap test in the model of eating problems as a mediator between perceived stress and depression were obtained as 0.13 and 0.03, respectively, due to the same sign of these Two limits, the path of perceived stress to depression with the mediation of eating problems is significant with a standard coefficient of 0.08 at the *p* < 0.05 level.

In response to the first research question, it can be said that eating problems can play a mediating role in the relationship between perceived stress and depression.

#### Question 2: Can sexual satisfaction play a mediating role in the relationship between depression in people with cancer and perceived stress?

**Figure 3:**
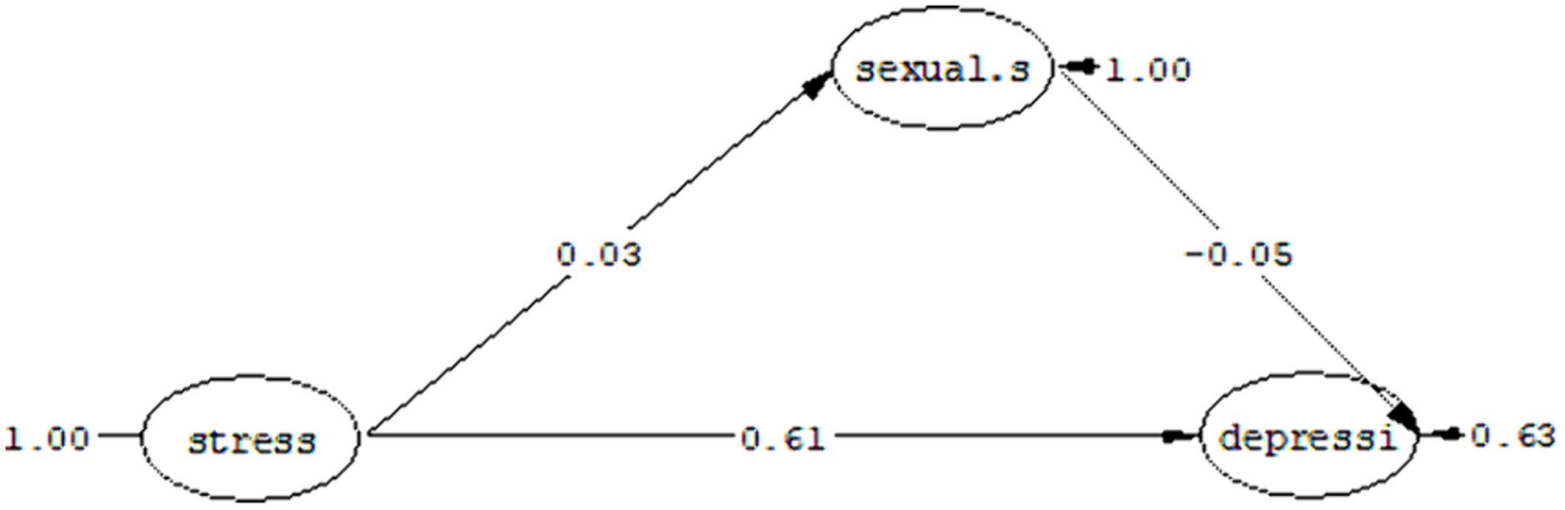
Mediation model of sexual satisfaction in the relationship between perceived stress and depression.

The fit indices as a result of the test of the proposed model are listed in Table 7, which shows that the proposed model has a good fit. As you can see in Figure 3, perceived stress has a direct effect coefficient (*t* = −0.39, *ß* = –0.03) on sexual satisfaction and a direct effect coefficient (*t* = 7.15, *ß* = 0.61) has depression. Also, sexual satisfaction has a direct effect coefficient (*t* = −0.83, *ß* = –0.05) on depression. Considering that in the structural model, the significance of the path coefficient is determined by using the t value, if the *t* value is more than 1.96, the relationship between the two structures is significant.

**Table 7:** Fit indices of the mediation model of sexual satisfaction in the relationship between perceived stress and depression.

**Fit indices**	***X*** ^**2**^ **/df**	**CFI**	**IFI**	**NFI**	**NNFI**	**RFI**	**RMSEA**
	3.34	0.89	0.89	0/90	0.90	0.88	0.08

Table 8 shows the standard coefficients, limits of the Bootstrap test and the estimation error of the indirect path (the mediating role of sexual satisfaction in the relationship between perceived stress and depression).

**Table 8:** Indirect path coefficients for the mediation model of sexual satisfaction in the relationship between perceived stress and depression.

**Indirect path**	**Indirect Beta**	**Number of sample reproductions**	**Bootstrap Value**		**Estimation Error**
			**Highest**	**Lowest**	
Mediating role of sexual satisfaction in the relationship between perceived stress and depression	0.01	1,000	0.03	−0.03	0.02

As you can see in Table 8, the highest and lowest limits of the Bootstrap test in the mediation model of sexual satisfaction between perceived stress and depression were obtained at 0.03 and −0.03, respectively. Due to the non-significance of these two extremes, the path of perceived stress to depression with the mediation of sexual satisfaction is not significant with a standard coefficient of 0.01 at the *p* < 0.05 level.

In response to the second research question, it can be said: sexual satisfaction cannot play a mediating role in the relationship between perceived stress and depression.

#### Question 3: Can emotion regulation play a mediating role in the relationship between depression in people with cancer and perceived stress?

**Figure 4:**
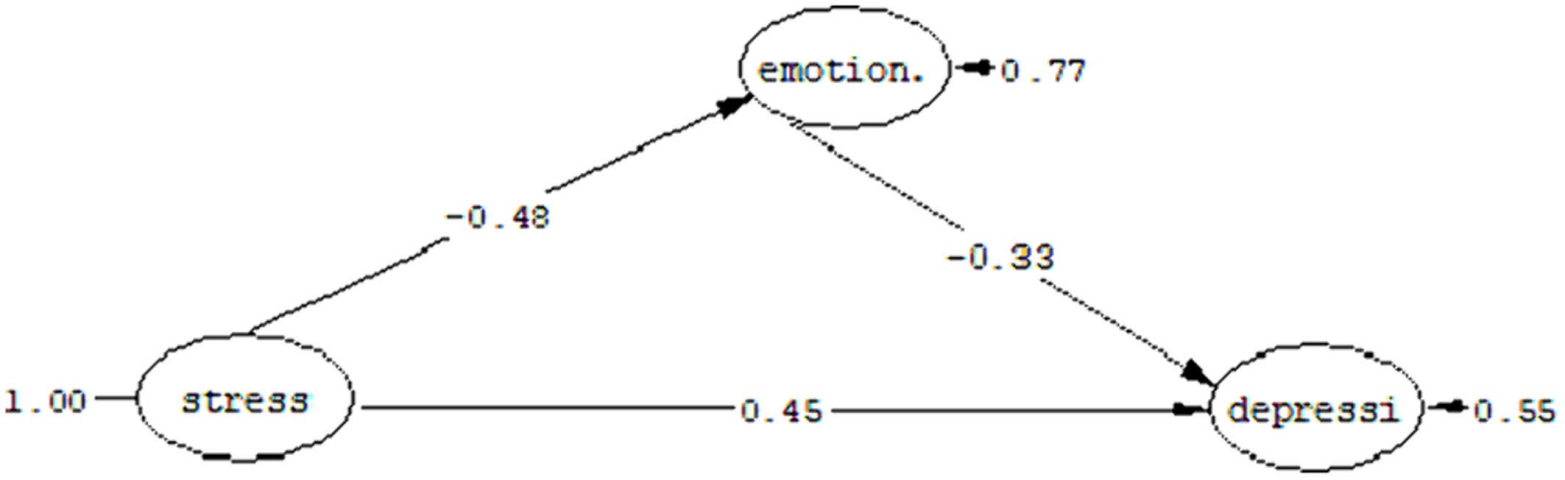
Mediation model of emotion regulation in the relationship between perceived stress and depression.

The fit indices as a result of the test of the proposed model are shown in Table 9, which shows that the proposed model has a good fit. As you can see in Figure 4, perceived stress has a direct effect coefficient (*t* = −4.64, *ß* = –0.48) on emotion regulation and a direct effect coefficient (*t* = −5.42, *ß* = –0.45) has depression. Likewise, emotion regulation has a direct effect coefficient (*t* = −3.52, *ß* = –0.33) on depression. Considering that in the structural model, the significance of the path coefficient is determined by using the t value, if the t value is more than 1.96, the relationship between the two structures is significant.

**Table 9:** fit indices of the mediation model of emotion regulation in the relationship between perceived stress and depression.

**Fit indices**	***X*** ^**2**^ **/df**	**CFI**	**IFI**	**NFI**	**NNFI**	**RFI**	**RMSEA**
	3.92	0.91	0.91	0/89	0.89	0.90	0.07

Table 10 shows the standard coefficients, limits of the Bootstrap test and the estimation error of the indirect path (the mediating role of emotion regulation in the relationship between perceived stress and depression).

**Table 10:** Indirect path coefficients for the mediation model of emotion regulation in the relationship between perceived stress and depression.

**Indirect path**	**Indirect Beta**	**Number of sample reproductions**	**Bootstrap Value**		**Estimation error**
			**Highest**	**Lowest**	
Mediating role of sexual satisfaction in the relationship between perceived stress and depression	0.04	1,000	0.09	0.02	0.03

As you can see in Table 10, the highest and lowest limits of the Bootstrap test in the mediation model of emotion regulation between perceived stress and depression were obtained as 0.09 and 0.02, respectively. The path of perceived stress to depression with the mediation of emotion regulation is significant with a standard coefficient of 0.01 at the *p* < 0.04 level.

In response to the third research question, it can be said that emotion regulation can play a mediating role in the relationship between perceived stress and depression.

#### Question 4: Can self-compassion play a mediating role in the relationship between depression in people with cancer and perceived stress?

**Figure 5:**
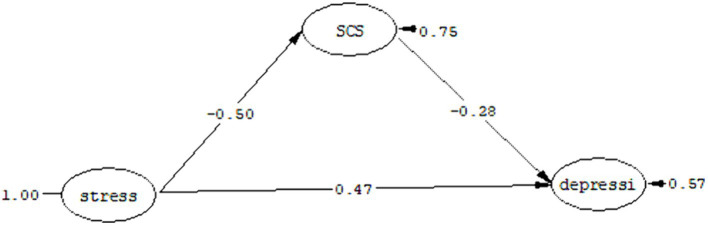
Mediation model of self-compassion in the relationship between perceived stress and depression.

The fit indices as a result of the test of the proposed model are listed in Table 11, which shows that the proposed model has a good fit. As you can see in Figure 5, perceived stress has a direct effect coefficient (*t* = −4.71, *ß* = –0.50) on self-compassion and a direct effect coefficient (*t* = 5.38, *ß* = 0.47) on depression. Also, self-compassion has a direct effect coefficient (*t* = −3.08, *ß* = –0.28) on depression. Considering that in the structural model, the significance of the path coefficient is determined by using the t value, if the t value is more than 1.96, the relationship between the two structures is significant.

**Table 11:** Fit indices of the mediation model of self-compassion in the relationship between perceived stress and depression.

Fit Indices	X^2^ /df	CFI	IFI	NFI	NNFI	RFI	RMSE A
	3.75	0.90	0.90	0/89	0.90	0.89	0.08

Table 12 shows the standardized coefficients, limits of the Bootstrap test and the estimation error of the indirect path (the mediating role of self-compassion in the relationship between perceived stress and depression).

**Table 12:** Indirect path coefficients for the mediation model of self-compassion in the relationship between perceived stress and depression.

**Indirect path**	**Indirect beta**	**Number of sample reproductions**	**Bootstrap value**		**Estimation error**
			**Highest**	**Lowest**	
Mediating role of self-compassion in the relationship between perceived stress and depression	0.12	1,000	0.19	0.04	0.04

As you can see in Table 12, the highest and lowest limits of the Bootstrap test in the mediation model of self-compassion between perceived stress and depression were obtained as 0.19 and 0.04, respectively, due to the co-sign of these two limits. The path of perceived stress to depression with the mediation of self-compassion is significant with a standard coefficient of 0.12 at the *p* < 0.05 level.

In response to the fourth research question, it can be said: self-compassion can play a mediating role in the relationship between perceived stress and depression.

#### Question 5: Does the assumed model of the research have a good fit or not?

**Figure 6:**
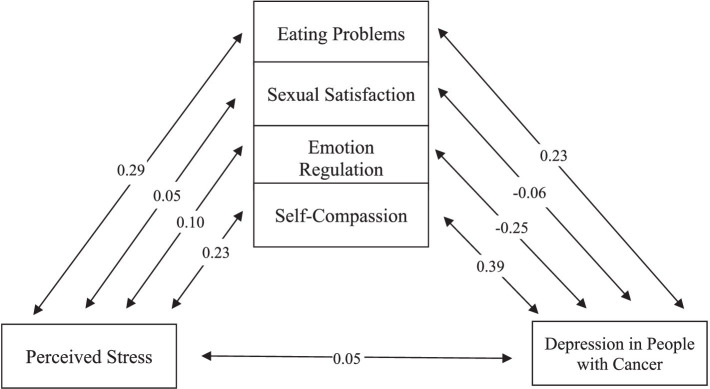
Assumed model.

The fit indices as a result of the test of the proposed model are listed in Table 13, which shows that the proposed model has a relatively favorable fit. As you can see in Figure 6, perceived stress has a direct effect coefficient (*t* = 4.33, *ß* = 0.29) on eating problems, direct coefficient (*t* = 0.71, *ß* = 0.05) on Sexual satisfaction, direct coefficient (*t* = −1.30, *ß* = –0.10) on emotion regulation, direct coefficient (*t* = −3.32, *ß* = –0.23) on self-compassion and direct effect coefficient (*t* = 0.47, *ß* = 0.05) on depression. Eating problems have a direct effect coefficient (*t* = 1.82, *ß* = 0.12) on depression. Sexual satisfaction has a direct effect coefficient (*t* = −1.07, *ß* = –0.06) on depression. Emotion regulation has a direct effect coefficient (*t* = −4.11, *ß* = –0.25) on depression. Self-compassion has a direct effect coefficient (*t* = −6.06, *ß* = –0.39) on depression. Considering that in the structural model, the significance of the path coefficient is determined by using the *t*-value, if the *t-*value is more than 1.96, the relationship between the two structures is significant.

**Table 13:** Fit indices of the assumed research model.

Fit indices	*X*^2^/df	CFI	IFI	NFI	NNFI	RFI	RMSE A
	4.19	0.86	0.86	0/89	0.87	0.85	0.09

## Discussion

The findings of the current study revealed a significant mediating role of eating problems in the relationship between perceived stress and depression. This aligns with previous studies conducted by Khoshnevis et al. (2012) and Sun et al. (2020) suggesting that cancer-related pressures and tensions can lead individuals to engage in harmful behaviors as a short-term coping mechanism. However, in the long run, these behaviors exacerbate the situation. When individuals perceive stress negatively, the likelihood of developing eating problems increases (Khoshnevis et al., 2012). Highly distressing and uncomfortable situations motivated by perceived stress prompt individuals to seek immediate relief. Unfortunately, especially when individuals lack the self-confidence to effectively manage stress, avoidance behaviors become activated. This avoidance, aimed at reducing pain and alleviating suffering, ultimately gives rise to problematic eating behaviors (Sun et al., 2020).

Contrary to expectations, sexual satisfaction was found to not play a mediating role in the relationship between perceived stress and depression. This finding contradicts the research conducted by Molavi et al. (2015), Zahniser and Conley (2018), and Lin et al. (2022). Cancer patients often experience a multitude of challenges that negatively impact their quality of life and sexual performance, such as the psychological and emotional effects associated with the disease and its treatment measures, as well as stress (Zahniser and Conley, 2018). Due to the various consequences of the disease, cancer patients’ perception of their physical attractiveness undergoes a change, leading them to no longer view themselves as physically and sexually attractive. Consequently, this cognitive shift results in reduced sexual satisfaction compared to their previous experiences (Molavi et al., 2015).

However, several factors may explain the lack of significance in the role of sexual satisfaction in the present study. The broad age range of the sample, coupled with the taboo nature of sexual topics in our society, and individuals’ reluctance to disclose issues in this realm, despite the researcher’s emphasis on confidentiality, may have contributed to inconsistent findings. Furthermore, cultural and ethnic influences on sexual activity and performance might have limited the information gathered by the questionnaire, consequently impacting the research outcomes.

On the other hand, the current study’s results indicate the mediating role of emotional regulation in the relationship between perceived stress and depression, consistent with previous research by Azizi and Mohamadi (2015), Mahin Islami Shahrbabaki (2015), Boyle et al. (2017), and Teng et al. (2022). Cancer challenges individuals’ perception of life as a structured and continuous process, which can have profound psychological consequences. Pain, suffering, concerns for the future of family members, fear of death, treatment complications, functional decline, disruption in body image, and sexual problems all contribute to the mental distress experienced by cancer patients. The crises brought on by cancer disrupt the harmony of mind, body, and psyche (Mahin Islami Shahrbabaki, 2015). Moreover, cancer patients go through various emotional reactions, such as shock, fear, disbelief, despair, anxiety, depression, and anger, toward themselves or the disease. These reactions are accompanied by a loss of control, autonomy, physical and social integration, and can lead to feelings of loneliness and guilt. Each of these factors can disrupt emotional control and expression, ultimately paving the way for mental disorders (Azizi and Mohamadi, 2015).

The cognitive strategies employed by cancer patients to regulate their emotions play a significant role in determining their overall health. Individuals who adopt adaptive emotion regulation strategies in the face of stressful events report lower levels of depression and, consequently, a higher quality of life. The negative relationship between adaptive strategies and depression can be attributed to the fact that these strategies allow individuals to view negative events from a different perspective, focusing on the positive aspects and potential benefits in the long run. As a result, they experience less distress and tension, effectively coping with the situation (Teng et al., 2022).

The findings of this study suggest that self-compassion plays a crucial role in mediating the relationship between perceived stress and depression. These findings are consistent with previous studies conducted by Sharifi Saki et al. (2018), Zhu et al. (2019), Narimani and Eyni (2020), and Williamson et al. (2022). One possible explanation for this finding is that individuals with cancer often experience a multitude of negative emotions. To cope with these emotions, it becomes necessary to find relief and resolution, ultimately overcoming these negative experiences (Narimani et al., 2020). Self-compassion serves as a potential response to personal suffering and failure, encompassing a protective and understanding attitude toward oneself while accepting limitations as a natural part of the human experience. By fostering self-compassion, individuals are able to create caring and compassionate inner processes, leading to positive changes that can be viewed as a physiological-psychological-neural treatment, thus inversely related to depression (Zhu et al., 2019).

Another explanation for these findings is that individuals with higher levels of self-compassion tend to experience fewer negative emotions when faced with unpleasant events, recognizing that illness and challenges are part of the human condition. As most negative emotions stem from ruminations following negative experiences, practicing mindfulness can reduce these emotions by minimizing rumination (Williamson et al., 2022).

In summary, the research findings unveil that eating problems play a significant mediating role in the intricate connection between perceived stress and depression among individuals with cancer, aligning with earlier studies. Contrarily, sexual satisfaction does not emerge as a mediator, contrary to expectations, potentially influenced by various factors such as societal taboos, reluctance to disclose, and cultural influences. The study confirms the mediating impact of emotional regulation, emphasizing the crucial role it plays in mitigating the psychological consequences of cancer-related challenges. Similarly, self-compassion emerges as a crucial mediator, aligning with previous research, as it acts as a protective response to personal suffering and failure. The comprehensive model, incorporating the mediating effects of eating problems, emotional regulation, and self-compassion, demonstrates a good fit in explaining the relationship between depression and perceived stress among individuals with cancer. Overall, these findings underscore the multifaceted influences on mental well-being in the context of cancer, providing valuable insights for tailored interventions to alleviate distress and enhance overall quality of life for affected individuals.

### Research limitations

Despite the valuable insights gained from this research, it is essential to acknowledge its limitations. Firstly, the study sample was limited to individuals with cancer undergoing treatment in Tehran’s hospitals and clinics, which may limit the generalizability of the results. Secondly, as a cross-sectional study, it cannot establish causal mechanisms. Lastly, the study lacked control over external validity-threatening factors, such as participant fatigue.

### Research suggestions

In light of these limitations, several suggestions for future research are proposed. Firstly, in addition to questionnaires, future studies should incorporate methods such as structured or semi-structured interviews for data collection. Furthermore, investigating the relationship between depression and perceived stress, with the mediation of eating problems, sexual satisfaction, emotional regulation, and self-compassion, should be conducted in a larger sample size and compared to the present study. Longitudinal studies would also be beneficial in clarifying the causal mechanisms between depression and perceived stress in individuals with cancer.

### Practical suggestions

Turning to practical suggestions, it is recommended to include emotion regulation skills and the application of emotion regulation strategies in the treatment of cancer patients to reduce symptoms of depression. Additionally, considering the hardships faced by cancer patients, emphasizing the importance of self-compassion and teaching methods to strengthen it can contribute to their psychological well-being. To address the impact of perceived stress on depression levels in cancer patients, stress management training courses should be incorporated into their schedule. Finally, training workshops on self-compassion and emotional regulation for cancer specialists and therapists can enhance their awareness and help improve treatment outcomes, symptom management, and overall quality of life for patients.

## Data availability statement

The raw data supporting the conclusions of this article will be made available by the authors, without undue reservation.

## Ethics statement

The studies involving humans were approved by Iran university of medical sciences. The studies were conducted in accordance with the local legislation and institutional requirements. The participants provided their written informed consent to participate in this study.

## Author contributions

RM: Conceptualization, Investigation, Software, Writing – original draft, Writing – review & editing. BG: Supervision, Writing – review & editing. KZ: Supervision, Writing – review & editing.

## References

[ref1] Abdollahi A. Taheri A. Allen K. A. (2020). Self-compassion moderates the perceived stress and self-care behaviors link in women with breast cancer. Psycho-Oncology 29, 927–933. doi: 10.1002/pon.5369, PMID: 32100897

[ref2] Alagizy H. Soltan M. Soliman S. Hegazy N. Gohar S. (2020). Anxiety, depression and perceived stress among breast cancer patients: single institute experience. Middle East Curr. Psychiatry 27, 1–10. doi: 10.1186/s43045-020-00036-x

[ref3] Asberg K. K. Wagaman A. (2010). Emotion regulation abilities and perceived stress as predictors of negative body image and problematic eating behaviors in emerging adults. Am. J. Psychol. Res. 6, 193–2017.

[ref4] Asghari F. Ghasemi Jobaneh R. Yousefi N. Saadat S. Rafiei G. F. (2017). Role of perceived stress and coping styles on the eating disorders of high school students of Rasht city in 2013. Commun. Health J. 8, 28–38.

[ref5] Azizi A. M. F. Mohamadi J. (2015). Comparison of personality factors and cognitive emotional regulation in gastric and lung cancer patients and normal subjects. Razi J. Med. Sci. 22, 1–9.

[ref6] Bahrami N. Yaghoob Zadeh A. Sharif Nia H. Soliemani M. A. Haghdoost A. A. (2016). Validity and reliability of the persian version of Larson sexual satisfaction questionnaire in couples. J. Kerman Univ. Med. Sci. 23, 344–356.

[ref7] Beck A. T. Epstein N. Brown G. Steer R. A. (1988). An inventory for measuring clinical anxiety: psychometric properties. J. Consult. Clin. Psychol. 56, 893–897. doi: 10.1037/0022-006X.56.6.893 3204199

[ref8] Bigdeli I. Najafy M. Rostami M. (2013). The relation of attachment styles, emotion regulation, and resilience to well-being among students of medical sciences. Iran. J. Med. Educ. 13, 721–729.

[ref9] Boyle C. C. Stanton A. L. Ganz P. A. Crespi C. M. Bower J. E. (2017). Improvements in emotion regulation following mindfulness meditation: effects on depressive symptoms and perceived stress in younger breast cancer survivors. J. Consult. Clin. Psychol. 85, 397–402. doi: 10.1037/ccp0000186, PMID: 28230391 PMC5364040

[ref10] Bray F. Ferlay J. Soerjomataram I. Siegel R. L. Torre L. A. Jemal A. (2018). Global cancer statistics 2018: GLOBOCAN estimates of incidence and mortality worldwide for 36 cancers in 185 countries. CA Cancer J. Clin. 68, 394–424. doi: 10.3322/caac.21492, PMID: 30207593

[ref11] Cohen S. Kamarck T. Mermelstein R. (1983). A global measure of perceived stress. J. Health Soc. Behav. 24:385. doi: 10.2307/2136404 6668417

[ref12] Cristóbal-Narváez P. Haro J. M. Koyanagi A. (2022). Longitudinal association between perceived stress and depression among community-dwelling older adults: findings from the Irish longitudinal study on ageing. J. Affect. Disord. 299, 457–462. doi: 10.1016/j.jad.2021.12.041, PMID: 34942218

[ref13] Din H. M. Akahbar S. A. N. Ibrahim R. (2019). The association between depression and sexual satisfaction among Malay elderly in Malaysia. Heliyon 5:e01940. doi: 10.1016/j.heliyon.2019.e01940 31338454 PMC6579850

[ref14] Ghassemzadeh H. Mojtabai R. Karamghadiri N. Ebrahimkhani N. (2005). Psychometric properties of a Persian-language version of the Beck depression inventory-second edition: BDI-II-PERSIAN. Depress. Anxiety 21, 185–192. doi: 10.1002/da.20070, PMID: 16075452

[ref15] Grønli J. Murison R. Fiske E. Bjorvatn B. Sørensen E. Portas C. M.. (2005). Effects of chronic mild stress on sexual behavior, locomotor activity and consumption of sucrose and saccharine solutions. Physiol. Behav. 84, 571–577. doi: 10.1016/j.physbeh.2005.02.007, PMID: 15811392

[ref16] Gross J. J. John O. P. (2003). Individual differences in two emotion regulation processes: implications for affect, relationships, and well-being. J. Pers. Soc. Psychol. 85, 348–362. doi: 10.1037/0022-3514.85.2.348, PMID: 12916575

[ref17] Hamilton L. D. Meston C. M. (2013). Chronic stress and sexual function in women. J. Sex. Med. 10, 2443–2454. doi: 10.1111/jsm.12249, PMID: 23841462 PMC4199300

[ref18] Homan K. J. Sirois F. M. (2017). Self-compassion and physical health: exploring the roles of perceived stress and health-promoting behaviors. Health Psychol. Open. 4:2055102917729542. doi: 10.1177/2055102917729542, PMID: 29379620 PMC5779931

[ref19] Hudson W. W. Harrison D. F. Crosscup P. C. (1981). A short-form scale to measure sexual discord in dyadic relationships. J. Sex Res. 17, 157–174. doi: 10.1080/00224498109551110

[ref20] Hur H.-K. Song H.-Y. (2008). Comparison of effects of perceived stress and coping patterns on depression between cancer patients and healthy adults. J. Hospice Palliative Care 11, 91–98.

[ref21] Joormann J. Stanton C. H. (2016). Examining emotion regulation in depression: a review and future directions. Behav. Res. Ther. 86, 35–49. doi: 10.1016/j.brat.2016.07.007, PMID: 27492851

[ref22] Khanjani S. Foroughi A. A. Sadghi K. Bahrainian S. A. (2016). Psychometric properties of Iranian version of self-compassion scale (short form). Pajoohandeh J. 21, 282–289.

[ref23] Khoshnevis N. Shahid Sales S. Alizadeh M. MirSadraei M. (2012). Akbari, Mohammad Esmaeil. Nutritional assessment of cancer patients by PG-SGA questionnaire in Cancer research center (CRC) of Shahid Beheshti University of Medical Sciences, Tehran, Iran, 2010. Pejouhesh Pezeshki 36, 132–138.

[ref24] Kissane D. W. Grabsch B. Love A. Clarke D. M. Bloch S. Smith G. C. (2004). Psychiatric disorder in women with early stage and advanced breast cancer: a comparative analysis. Aust. N. Z. J. Psychiatry 38, 320–326. doi: 10.1080/j.1440-1614.2004.01358.x, PMID: 15144508

[ref25] Kline R. B. (2023). Principles and practice of structural equation modeling Guilford Publications.

[ref26] Larson J. H. Anderson S. M. Holman T. B. Niemann B. K. (1998). A longitudinal study of the effects of premarital communication, relationship stability, and self-esteem on sexual satisfaction in the first year of marriage. J. Sex Marital Ther. 24, 193–206. doi: 10.1080/00926239808404933, PMID: 9670124

[ref27] Lee J. S. Joo E. J. Choi K. S. (2013). Perceived stress and self-esteem mediate the effects of work-related stress on depression. Stress. Health 29, 75–81. doi: 10.1002/smi.2428, PMID: 22610597

[ref28] Li Y. Yang Y. Zhang R. Yao K. Liu Z. (2015). The mediating role of mental adjustment in the relationship between perceived stress and depressive symptoms in hematological cancer patients: a cross-sectional study. PLoS One 10:e0142913. doi: 10.1371/journal.pone.0142913, PMID: 26587991 PMC4666411

[ref29] Lin H. Fu H.-C. Wu C.-H. Tsai Y.-J. Chou Y.-J. Shih C.-M.. (2022). Evaluation of sexual dysfunction in gynecologic cancer survivors using DSM-5 diagnostic criteria. BMC Womens Health 22, 1–7. doi: 10.1186/s12905-021-01559-z, PMID: 34986812 PMC8734329

[ref30] Mahin Islami Shahrbabaki MH. Effectiveness of the training of emotion regulation strategies on children with cancer. Third international conference of psychology and sociology (2015).

[ref31] Miovic M. Block S. (2007). Psychiatric disorders in advanced cancer. Cancer 110, 1665–1676. doi: 10.1002/cncr.22980 17847017

[ref32] Mischoulon D. Eddy K. T. Keshaviah A. Dinescu D. Ross S. L. Kass A. E.. (2011). Depression and eating disorders: treatment and course. J. Affect. Disord. 130, 470–477. doi: 10.1016/j.jad.2010.10.043, PMID: 21109307 PMC3085695

[ref33] Molavi A. Hekmat K. Afshari P. Hoseini M. (2015). Evaluation of couples' sexual function and satisfaction after mastectomy. Iran. J. Obstetrics Gynecol. Infertility 17, 17–24. doi: 10.22038/ijogi.2015.3700

[ref34] Narimani M. Eyni S. (2020). Relationship between meaning of life, self- compassion and sense of coherence with perceived stress in cancer patients. Iran. J. Cancer Care. 1, 1–10. doi: 10.29252/ijca.1.3.1

[ref35] Narimani M. Ghasemkhanlo A. Sabri V. (2020). The comparison of self-compassion, anxiety and depression inpatients with chronic pain and normal people. Anesthesiol. Pain. 11, 57–65.

[ref36] Peleg-Sagy T. Shahar G. (2013). The prospective associations between depression and sexual satisfaction among female medical students. J. Sex. Med. 10, 1737–1743. doi: 10.1111/jsm.12176, PMID: 23651294

[ref37] Raes F. Pommier E. Neff K. D. Van Gucht D. (2011). Construction and factorial validation of a short form of the self-compassion scale. Clin. Psychol. Psychother. 18, 250–255. doi: 10.1002/cpp.702, PMID: 21584907

[ref38] Roshandel G. Ferlay J. Ghanbari-Motlagh A. Partovipour E. Salavati F. Aryan K.. (2021). Cancer in Iran 2008 to 2025: recent incidence trends and short-term predictions of the future burden. Int. J. Cancer 149, 594–605. doi: 10.1002/ijc.33574, PMID: 33884608

[ref39] Roy S. Jahan K. Alam N. Rois R. Ferdaus A. Israt S.. (2021). Perceived stress, eating behavior, and overweight and obesity among urban adolescents. J. Health Popul. Nutr. 40, 1–13. doi: 10.1186/s41043-021-00279-2 34920764 PMC8679564

[ref40] Saeedi N. R. Sharbaf H. A. Ebrahimabad M. J. A. Kareshki H. (2019). Psychological consequences of breast cancer in Iran: a meta-analysis. Iran. J. Public Health 48:816. doi: 10.18502/ijph.v48i5.1796 31523637 PMC6717424

[ref41] Safaei M. Shokri O. (2014). Assessing stress in cancer patients: factorial validity of the perceived stress scale in Iran. Iran. J. Psychiatric Nurs. 2, 13–22.

[ref42] Sajadi Hezaveh Z. Golafrouz H. Piran A. Vafa M. (2023). Validity and reliability of the persian version of the council on nutrition appetite questionnaire and its simplified version in iranian community-dwelling older adults. JNFS 8, 163–171.

[ref43] Sartorius N. Holt R. I. Maj M. (2014). Comorbidity of mental and physical disorders Karger Medical and Scientific Publishers.

[ref44] Sharifi Saki S. Alipour A. AghaYousefi A. R. Mohammadi M. R. Ghobari Bonab B. Anbiaee R. (2018). Relationship of patience and self- compassion with depression in patients with breast Cancer. Iran. J. Breast Diseases 11, 36–45. doi: 10.30699/acadpub.ijbd.11.2.36

[ref45] Sun H. Sudip T. Fu X. Wen S. Liu H. Yu S. (2020). Cachexia is associated with depression, anxiety and quality of life in cancer patients. BMJ Support. Palliat. Care 13, e129–e135. doi: 10.1136/bmjspcare-2019-002176 32917649

[ref46] Tavares I. M. Schlagintweit H. E. Nobre P. J. Rosen N. O. (2019). Sexual well-being and perceived stress in couples transitioning to parenthood: a dyadic analysis. Int. J. Clin. Health Psychol. 19, 198–208. doi: 10.1016/j.ijchp.2019.07.004, PMID: 31516498 PMC6732775

[ref47] Teng S. Wang M. Han B. Ma Y. Du H. Ji L.. (2022). The relationship between post-traumatic stress and negative emotions in patients with breast cancer: the mediating role of emotion regulation. J. Psychosoc. Oncol. 40, 506–518. doi: 10.1080/07347332.2021.1950885 34392806

[ref48] Williamson T. J. Garon E. B. Shapiro J. R. Chavira D. A. Goldman J. W. Stanton A. L. (2022). Facets of stigma, self-compassion, and health-related adjustment to lung cancer: a longitudinal study. Health Psychol. 41, 301–310. doi: 10.1037/hea0001156, PMID: 35324247 PMC9030259

[ref49] Wilson M.-M. G. Thomas D. R. Rubenstein L. Z. Chibnall J. T. Anderson S. Baxi A.. (2005). Appetite assessment: simple appetite questionnaire predicts weight loss in community-dwelling adults and nursing home residents. Am. J. Clin. Nutr. 82, 1074–1081. doi: 10.1093/ajcn/82.5.1074, PMID: 16280441

[ref50] Zahniser E. Conley C. S. (2018). Interactions of emotion regulation and perceived stress in predicting emerging adults’ subsequent internalizing symptoms. Motiv. Emot. 42, 763–773. doi: 10.1007/s11031-018-9696-0

[ref51] Zhu L. Yao J. Wang J. Wu L. Gao Y. Xie J.. (2019). The predictive role of self-compassion in cancer patients' symptoms of depression, anxiety, and fatigue: a longitudinal study. Psycho-Oncology 28, 1918–1925. doi: 10.1002/pon.5174, PMID: 31291695

